# Neighborhood Walkability and Active Transportation: A Correlation Study in Leisure and Shopping Purposes

**DOI:** 10.3390/ijerph17072178

**Published:** 2020-03-25

**Authors:** Eun Jung Kim, Jiyeong Kim, Hyunjung Kim

**Affiliations:** 1 Department of Urban Planning, Keimyung University, 1095 Dalgubeol-daero, Dalseo-gu, Daegu 42601, Korea; kimej@kmu.ac.kr (E.J.K.); th154@naver.com (J.K.); 2 Department of Civil and Environmental Engineering, Seoul National University, Gwanak-ro 1, Gwanak-gu, Seoul 08826, Korea

**Keywords:** active transportation, walking, cycling, leisure trip, shopping trip, Walk Score, Walkability Score, multilevel logistic regression model, Seoul

## Abstract

A walkable environment is a crucial factor for promoting active transportation. The purpose of this study is to examine the association between neighborhood walkability and active transportation for noncommuting purposes (leisure and shopping) in Seoul, Korea. The Walkability Score is used as a measure of walkability, and a multilevel logistic regression model is employed to measure the odds of active transportation (i.e., walking and cycling; nonmotorized trips) at two levels: individual (level 1) and neighborhood (level 2). The results of the study showed that the Walkability Score was significantly correlated with higher odds of active transportation in shopping models. Specifically, every one-point increase in the Walkability Score was associated with 1.5%–1.8% higher odds of active transportation in shopping models. However, there was no significant correlation between the two in leisure models. Meanwhile, individual characteristics associated with the odds of active transportation differed in the leisure and shopping models. Older age was positively correlated with the odds of active transportation in the leisure model, while females showed a positive correlation in the shopping model. Based on the study, urban and transportation planners can recommend urban policies to promote active transportation in an urban setting.

## 1. Introduction

An increase in sedentary behavior and a proportionate growth in chronic diseases have been considered the most critical public health issues in the modern world, prompting several researchers in the public health and urban planning fields to investigate the environmental impact of promoting higher levels of physical activity [1,2]. As a means to promote physical activity, walking and cycling can be considered feasible daily activities for most people [3,4,5]. Among the different types of physical activities, walking and cycling are considered suitable for all age groups, given that they do not require special skills or facilities, and allow people to manage the intensity of their own movements [5]. Therefore, efforts to enhance walking and cycling within the community have been gaining momentum lately [5].

Promoting physical activity can help prevent a rise in the overweight and obese population and reduce the risk of potential chronic ailments such as respiratory diseases and Type 2 diabetes, as well as mortality risk from cardiovascular diseases and cancer [3,6,7,8,9,10]. Enhanced physical activity can also benefit mental health as it can improve emotions and the sense of recognition [11], reduce pressure [12,13], and depression [14]. From the urban and transportation planning perspective, walking and cycling, usually termed as “nonmotorized transport” or “active transportation,” can be considered an important means for promoting sustainable cities and for providing social, environmental, and economic benefits [4,15]. Therefore, there is a growing emphasis on the importance of active transportation in light of urban problems such as traffic congestion, environmental pollution, energy shortage, and an increase in the obese population.

It is imperative to promote environmental and policy approaches to encourage physical activity considering that it can benefit all citizens in the neighborhood [16,17,18]. Accordingly, efforts to find an adequate built environment for physical activity, especially a walkable environment, has attracted the attention of policymakers, urban and transportation researchers, and public health scientists. Several studies have identified built environmental factors that most significantly influence walking behavior in urban and suburban areas [4,7,19,20,21], and efforts were made to develop a methodology to objectively measure the level of walkability. Consequently, several indices such as the Walk Score, Walkability Index, and Pedestrian Index of the Environment were developed combining various built environmental variables that influence walking behavior [7,19,20,21,22,23,24,25,26,27,28,29,30,31,32]. The Walk Score is one of the popular indices that objectively measures neighborhood walkability, taking into account the accessibility of amenities in the vicinity (e.g., grocery stores, restaurants, shopping centers, coffee shops, parks, schools) and pedestrian friendliness (e.g., intersection density and average block length) [25], and is currently used in various fields, including public health, real estate, and urban planning [33,34,35,36,37]. Studies have verified whether the Walk Score is appropriate to describe the level of walkability, and correspondingly, several works of the literature showed that a higher level of Walk Score is positively correlated with walking behavior [7,26,29,30,38,39,40,41,42].

A number of studies have investigated the association between walkability and health indicators using the Walk Score [42,43,44]. Xu and Wang examined the impact of the neighborhood environment on physical inactivity and obesity in Washington, D.C. in the United States. Using a multilevel regression model, they found that street connectivity was negatively associated with obesity, while Walk Score was negatively associated with physical inactivity. They also found that the obesity risk varied depending on urbanicity levels and gender, with a higher Walk Score linked to a lower risk of obesity in urban areas for females [43]. Wasfi et al. examined the influence of neighborhood walkability on the Body Mass Index (BMI) of urban Canadians using the Walk Score and the National Population Health Survey of Canada, and found that neighborhood walkability influences BMI trajectories for males [42]. McCormack et al. explored the relationship between walkability and waist circumference, waist-to-hip ratio, and BMI in Calgary, Alberta, Canada. Correspondingly, they found that a higher Walk Score was associated with lower odds of having a large waist circumference; neighborhoods with a lower Walk Score had higher odds of a high waist circumference, BMI, and waist circumference–BMI risk [44]. In short, previous studies have shown that developing a walkable environment can influence physical activity, which can lead to a significant population health impact.

## 2. Research Background

Over the past several decades, rapid industrialization and urbanization have led to significant lifestyle changes resulting in an increasing overweight and obese population, creating a significant public health burden in many countries [5,45]. A practical way to encourage physical activity on a daily basis is to promote active transportation. Therefore, several studies identified that a walkable environment has a positive association with active transportation [7,21,26,27,28,29,30,31,32]. For example, Reyer et al. used the Walkability Index and the Walk Score to explore the link to active transportation and found a tendency toward more active travel in more walkable neighborhoods [7]. Likewise, Knuiman et al. examined the relationship of neighborhood walkability and accessibility to a destination using walking as transportation in Perth, Australia. They found that accessibility to local destination, land use mix, and street connectivity are important determinants for promoting walking as a means of transportation [32].

Studies regarding the relationship between walkability and noncommuting active trips mainly considered the leisure purpose of walking or cycling. Investigating the neighborhood walkability in Canada, de Sa and Arden found that respondents living in highly walkable 500 m buffer zones (upper quartiles of the walkability index) were more likely to walk or cycle for leisure than those living in low-walkable buffer zones. When a 1000 m buffer zone was applied, respondents in more walkable neighborhoods were more likely to walk or cycle for both leisure and transport-related purposes [27]. Dyck et al. examined the association between leisure-time physical activity and perceived neighborhood environmental walkability in Belgium, Australia, and the United States. Except for the city of Ghent, Belgium, there was a positive linear association with recreational walking and leisure time physical activity [28]. Thielman et al. estimated the association between walkability and physical activity by transport walking and leisure time physical activity in Canada at the national level. They found that walkability was associated with transport walking in all age groups and city sizes. However, it had an inverse association with leisure-time physical activity among young adults and in large cities [29]. Some studies examined walkability for shopping purposes [21,30,31], but there are few studies that examined both leisure and shopping trips depending on the neighborhood walkability. For example, Habibian and Hosseinzadeh examined walkability across trip purposes including commuting, educational, and shopping, although they did not consider trips for leisure [31]. Manaugh and El-Geneidy examined the relationship between the trip purpose and walkability in nonmotorized mode of transportation; however, they considered noncommuting trip purposes mainly for shopping [30]. Lefebvre-Ropars et al. examined the association between walking time and the built environment using the Pedestrian Index of the Environment (PIE). They considered both shopping and leisure as noncommuting trips, and found that the PIE was more strongly correlated to the choice of walking for work, leisure, or shopping in very short trips [21]. However, they did not consider cycling as active transportation. In sum, previous studies on noncommuting active trips mainly focused on leisure walking/cycling, or investigated either leisure or shopping trips, or mainly focused on walking.

Korea is facing a severe health problem related to physical inactivity [45]. According to the Community Health Survey of Korea, the walking rate (the percentage of people who walked more than five days a week for more than 30 min a day in a week) in the country decreased overall during the past decade, from 50.6% in 2008 to 42.9% in 2018, while the proportion of individuals who are obese increased from 21.6% in 2008 to 31.8% in 2018 [46]. This increase in physical inactivity and obesity can be considered a social problem in Korea, which underscores the importance of developing pedestrian-friendly urban environments [47,48,49,50]. Accordingly, the city of Seoul is promoting “Walkable City, Seoul” as a major urban policy to enhance citizens’ health and reduce traffic congestion [51]. A pedestrian-oriented traffic environment is being modeled with increasing safe walking zones and connecting touristic spots. Furthermore, bicycling is being promoted by expanding bike-related infrastructure, such as a sharing bike system.

A recent study by Kim et al. examined the association between walkability and active commuting (i.e., walking and cycling to work or school) in Seoul using the Walkability Score, and empirically found a positive correlation between walkability and active commuting [52]. This raises another question as to whether walkability will also have a significant positive association in the case of noncommuting trips (e.g., leisure and shopping trips) with active transportation (i.e., walking and cycling). In addition, based on trip purposes, would a walkable environment associate similarly or differently within leisure and shopping trips? Especially, noncommuting trips are an intentional trip that may more likely depend on the neighborhood environment than a commuting trip [30]. Some studies found that the distance and duration of noncommuting walking were substantially longer than they were for commuting purpose, as well as the importance of urban forms on noncommuting trips [21,53]. In particular, compared to commuting, a noncommuting trip can induce more walking and cycling if an adequate walkable and bikeable built environment is provided in the neighborhood. This study expands the scope of Kim et al.’s [52] research by considering the Walkability Score and active transportation differentiating the trip purposes to noncommuting. The purpose of this study is to examine the association between the level of walkability measured by the Walkability Score and active transportation for noncommuting trips in Seoul, Korea. Specifically, the relationship between the walkability level and active transportation (i.e., walking and cycling) in noncommuting trips (i.e., shopping and leisure trip) will be examined considering both individual characteristics and the built environment of the neighborhood by conducting a multilevel analysis in Seoul.

## 3. Materials and Methods

### 3.1. Study Area

**Figure 1:**
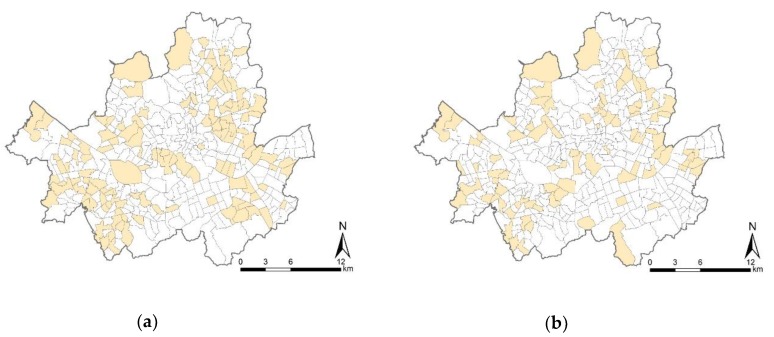
Study area: (**a**) Neighborhoods with at least 30 individual respondents for leisure trips (N = 129); (**b**) Neighborhoods with at least 30 individual respondents for shopping trips (N = 91).

The study focuses on the city of Seoul, and uses travel mode data from the Household Travel Diary Survey adapted from the Korea Transport Database [54]. The total number of home-based trips for leisure and shopping purposes in the survey were 9998 and 8578, respectively. The city of Seoul consists of 424 neighborhoods with their own administrative offices. The neighborhood is referred to as “dong” in Korea, which is the most disaggregated administrative unit. Of the 424 neighborhoods, some had very few sample numbers in the survey. For example, in the case of Jamwon-dong, only four individuals responded to the survey. Neighborhoods that had notably few samples such as Jamwon-dong were excluded from the multilevel modeling, given that ensuring a sufficient number of samples is one of the most important issues in multilevel analyses [55,56,57]. For unbiased results, some studies suggested ”30/30” and “20/50” rules that indicated the minimum number of observations per group/minimum number of groups. Kreft recommended the “30/30” [58], while Hox recommended the “‘20/50” rule [59]. To prevent biased estimate of parameters, we employed a minimum of 30 respondents per neighborhood for the study. Out of the 424 neighborhoods, the number of corresponding neighborhoods was 129 and 91 in the leisure and shopping models, respectively. Overall, the study used 5742 individuals nested within 129 neighborhoods and 3722 individuals nested within 91 neighborhoods in the leisure and shopping trip models, respectively, as shown in Figure 1.

### 3.2. Measures

As mentioned earlier, this research expands on a study by Kim et al. [52]. In this study, an investigation of the correlation between noncommuting active trips (i.e., leisure and shopping) and the Walkability Score was conducted. Accordingly, independent variables were basically derived from related data sources and methodology from Kim et al. [52], differentiating the trip purposes used as the dependent variables. The measurements and data sources of variables used in this study are shown in Table 1.

#### 3.2.1. Individual-level Variables (Level 1)

All individual-level variables of this study were acquired from the 2016 Household Travel Diary Survey from the Korea Transport Database [54], which is a traffic-related survey conducted every five years across the country and considered as a nationwide passenger survey in Korea. This survey examines the travel diary of household members aged five and older on weekdays, and is conducted through home visits and online using a self-reported questionnaire [60].

● Dependent variables: Travel mode (motorized vs. nonmotorized modes)

As dependent variables, this study used active transportation (e.g., walking and cycling) separately for leisure and shopping purposes. From the Household Travel Diary Survey, there are four travel modes—walking, bicycling, public transport, and private automobile. For the analysis, they were coded as binaries—1 for nonmotorized modes (walking and cycling) and 0 for motorized modes (public transport and private automobile).

● Individual socioeconomic status variable

At the individual level, several socioeconomic status variables were considered as confounding factors, including age, gender, household income, and car ownership. These individual variables were also acquired from the 2016 Household Travel Diary Survey from the Korea Transport Database [54].

#### 3.2.2. Neighborhood-level Variables (Level 2)

This study employed the neighborhood as a spatial unit for multilevel modeling at level 2. All neighborhood-level variables in this study used the mean values of each neighborhood.

● Walkability Score

As a key independent variable, this study employed the Walkability Score, which was assessed in Seoul by Kim et al. [61]. Moreover, a recent study found that the Walkability Score is a reliable index to measure environmental walkability in the city [62]. The value of the Walkability Score is calculated by the Walk Score algorithm, which basically calculates the accessibility of utilitarian destinations with a distance decay function. They include nine amenities essential to everyday life—grocery stores, restaurants, shopping centers, coffee shops, banks, parks, schools, books, and entertainment. The Walkability Score calculates the closest network distance from each amenity and then awards 100% of the maximum points to amenities located within a network distance of 400 m, 75% within 800 m, 40% within 1.2 km, and 12.5% within 1.6 km of a given location [61]. By combining the accessibility of nine types of utilitarian destinations, the scores are normalized on a scale of 0 to 100. Additionally, poor pedestrian friendliness is considered as a penalty element. It considers the intersection density and average block length as factors of pedestrian friendliness. Areas with lower intersection density (no penalty: intersections per square mile > 200) and longer average block length (no penalty: average block length < 120 m) receive penalties up to 10% of the total score. The Walk Score ranges from 0 (car-dependent) to 100 (walkers’ paradise) [25]. Geospatial data used for assessing the Walkability Score in Seoul were collected from both governmental websites and private companies. More detailed information on measures, data sources, and calculation methods is found in Kim et al. [61].

● Neighborhood environmental variables

The Walkability Score is adapted from the Walk Score, which is an index that combines various variables that represent urban form such as density, diversity, and destination accessibility [21,62]. Since the Walk Score itself is a composite index of neighborhood walkability, there are some studies that include only itself as a neighborhood environmental variable [7,63]. However, one study found that there are some urban form elements such as land use diversity and walking route supply that the Walk Score does not include [21]. Therefore, this study considered land use mix and sidewalk length as additional neighborhood environmental variables. Land use mix is an important urban environmental variable for promoting walking and cycling. Some studies have shown that the higher the land use mix, the more people walk and/or are physically active [31,32,64,65]. The entropy index was used to measure a land use mixture of residential, commercial, industrial, and greenspaces. It ranges from 0 (single use) to 1 (perfect mixing). Moreover, sidewalk length (i.e., total length of sidewalks per square kilometer) was considered in this study, given that it was positively associated with the minutes of neighborhood-based walking for transportation [66]. The data used in the analysis were obtained from the public source of the National Spatial Data Infrastructure Portal [67], while the neighborhood environmental variables were captured by ArcGIS.

### 3.3. Data Analysis

A multilevel logistic regression model was used, with the two odds of nonmotorized trips (walking and cycling) for (1) leisure purposes and (2) shopping purposes. An odds ratio (OR) is a statistic that quantified the association between an outcome and exposure. When calculating a logistic regression analysis, the regression coefficient is an estimated increase in the log probability of the outcome according to a unit increase in the exposure value [68]. If the OR is exactly 1, there is no correlation between outcome and exposure. When the OR is greater than 1, then there is a positive association, conversely if the OR is less than 1, then there is a negative correlation between outcome and exposure. Applying this concept here, this study examined the correlation between outcome (odds of nonmotorized trips for noncommuting) and exposure (individual and neighborhood variables). The multilevel data included two levels: individual (level 1) and neighborhood (level 2). Individuals living in the same neighborhood shared the same Walkability Score and the neighborhood environmental characteristics including land use mix and sidewalk length at the corresponding level. R software was used for the analysis in this study.

## 4. Results

### 4.1. Descriptive Statistics of Variables

As shown in Table 2, the proportions of individuals using nonmotorized modes of transportation for leisure and shopping purposes were 81.8% and 76.8%, respectively. In both the samples, the ratio of the motorized mode to nonmotorized mode was approximately 1 to 4. From the individual variables, the participants for the leisure and shopping trips reported a mean age of 61.1 and 53.5 years, respectively. The median income level of households per month was 4 and 3, respectively, for each subsample; the value of 3 corresponds to 2–3 million won while 4 corresponds to 3–5 million won. The variable of household income ranged from 1 (less than 1 million won) to 6 (more than 10 million won). In the leisure and shopping trip group, the proportion of female participants was 61.4% and 90.9%, and the proportion of car ownership was 54.8% and 63.3%, respectively.

The mean values of the neighborhood environmental variables were slightly different because the target areas (number of neighborhoods for leisure model = 129, number of neighborhoods for shopping model = 91) varied between leisure and shopping travels, although it was basically similar in the two samples. In the samples of leisure and shopping purposes, the mean value of Walkability Score was 67.55 (SD = 9.0) and 67.71 (SD = 9.8), respectively. Meanwhile, the mean value of land use mix was 0.52 (SD = 0.3) and 0.53 (SD = 0.3), and the mean value of sidewalk length was 1.99 (SD = 0.6) and 1.95 (SD = 0.6), respectively. Both variables of land use mix and sidewalk length were square root-transformed for use in the multilevel analysis.

### 4.2. Results of Multilevel Logistic Models: Odds of Active Transportation for Leisure and Shopping Purposes

This study conducted a multilevel logistic model because individual variables were nested within neighborhoods. Generally, there are three phases of procedure for multilevel logistic regression: (step 1) an empty model without predictors to assess variation of log-odds between clusters, (step 2) an intermediate model to assess the variation of the lower-level effects between clusters, and (step 3) a final model to test a research hypothesis [69]. Based on the procedure, this study first ran a model without predictors and found it was necessary to employ the multilevel analysis. In the second phase, we tested which variables were associated with the odds of outcome (active transportation) by performing a likelihood-ratio test. The final model was then presented according to theoretical importance as well as the statistical significance of variables. There are two final models each in the leisure and shopping models. Model 1 considers individual and neighborhood variables, but only considered the Walkability Score as a neighborhood variable, while Model 2 included additional neighborhood-level variables in Model 1. Specifically, the dependent variable is binary, for example, an individual using nonmotorized transportation (=1, and 0 otherwise) for leisure and shopping purposes. Model 1 included age, gender, income, and car ownership as individual variables (level 1) and the Walkability Score as a neighborhood variable (level 2). Meanwhile, Model 2 basically included all variables used in Model 1, but added land use mix and sidewalk length as neighborhood variables (level 2). The results of multilevel logistic models are shown in Table 3. Since Models L–2 and S–2 contained theoretically important neighborhood environmental variables such as land use mix and sidewalk length, the results from both Model 1 (L–1 and S–1) and Model 2 (L–2 and S–2) are reported in this section.

#### 4.2.1. Odds of Active Transportation for Leisure Purposes

The results of the analysis are as follows. First, the Walkability Score was insignificant in both Models L–1 and L–2. At the 0.1 significance level, there was no evidence of statistical interaction between the Walkability Score and the odds of nonmotorized trips in both Model L–1 (*p* = 0.145) and Model L–2 (*p* = 0.178). Second, age was positively associated with the odds of nonmotorized trips in this model. Older adults were more likely to use active transportation for leisure purposes. The results are similar to a previous study, which found that young adults had a negative association with leisure physical activity [29]. Third, car ownership was a statistically significant predictor of nonmotorized trips. Individuals having a car are much less likely to walk or cycle for leisure purpose. Fourth, gender and household income were not associated with the odds of nonmotorized trips in both the models. Previous studies also showed different associations on gender and the Walk Score (e.g., positive association of lower risk of obesity for females in urban areas [43], while showing no significant influence on BMI for females [42]). With respect to income level, a previous study showed that lower income individuals were more likely to walk and cycle for commuting purposes [52]. For economic reason, the lower the income, the more likely it is to commute by walking and cycling. On the other hand, because travel for leisure and shopping purposes are less sensitive to income than commuting travel, it is understandable that there no significant correlation between income level and the odds of nonmotorized trips in this study. Fifth, unlike previous studies [31,32,64,65,66], additional neighborhood environmental variables, including land use mix and sidewalk length, had no statistical relationships with the odds of nonmotorized trips in Model L–2.

#### 4.2.2. Odds of Active Transportation for Shopping Purposes

The results for the shopping model can be summarized as follows. First, similar to previous studies [30,31], the Walkability Score was significantly associated with the odds of nonmotorized trips in both Model S–1 and Model S–2. Every one-point increase in the Walkability Score was associated with 1.5% and 1.8% higher odds of nonmotorized trips in Model S–1 (OR: 1.015, 95% CI = 1.003–1.026) and Model S–2 (OR: 1.018, 95% CI = 1.005–1.031), respectively. Second, the female gender was positively correlated with the odds of nonmotorized trips in both models, as they tended to walk and cycle more for shopping purposes. Third, as with leisure models, car ownership was significantly associated with nonmotorized trips. Individuals with cars were associated with lower odds of nonmotorized trips for shopping. Fourth, there were contradictory results depending on individual variables. For example, age was a significant predictor in leisure models similar to the previous study [29]; however, it was insignificant in shopping models. Income level had no significance in both leisure and shopping models. Fifth, similar to leisure models, there were no significant correlations between the odds of nonmotorized trips and neighborhood variables such as land use mix and sidewalk length. Finally, according to the intraclass correlation coefficient (ICC) value, about 4.5% of the total variance in the odds of nonmotorized trips for shopping purposes was accounted for by differences of the Walkability Scores between neighborhoods in Model S–1. The ICC represents the proportion of the between-group variance compared to the total variance [70]. The interpretation of the ICC is the expected correlation between randomly selected observations from the same group [71]. In this study, the ICC is the proportion of variance in the odds of nonmotorized trips (outcome variable) that is explained by the neighborhood (level 2). It indicated that neighborhood walkability was one of the most important factors affecting individuals’ behavior of active transportation for shopping purposes. Model S–2 showed about 4.7% explanatory power of neighborhood variables (level 2), but the significance of additional neighborhood environmental variables, such as land use mix and sidewalk length, were not guaranteed. There are some arguments that if ICC is close to zero (typically less than 4%–5%), there is lesser need to use a multilevel analysis [72,73]. However, it is still necessary to use multilevel analysis in nested data [73].

## 5. Discussion

This study examined the correlation between the level of walkability and noncommuting trips by conducting multilevel logistic regression analysis with the two odds of active transportation (i.e., walking and cycling) for leisure and shopping purposes. It is an expansion of a prior study that revealed a positive correlation between neighborhood walkability and active commuting in Seoul [52], since it is likely that noncommuting trips are more influenced by the neighborhood environment than commuting trips when people walk or cycle [30]. As a result, we empirically discovered that the walkability level of Seoul’s neighborhoods was positively correlated with the probability of active transportation for shopping purposes while showing no statistical correlation in leisure purposes.

Discussions based on the findings are presented as follows. First, the correlation between the Walkability Score and the nonmotorized trips varied in the leisure and shopping models. In the shopping models, the Walkability Score was positively correlated with the odds of nonmotorized trips (Model S–1: OR = 1.015, 95% CI = 1.003–1.026; Model S–2: OR = 1.018, 95% CI = 1.005–1.031). However, there was no significant correlation between the Walkability Score and the odds of nonmotorized trips for leisure purposes. This differs from the findings of previous literature that showed that the odds ratios of walking for the Pedestrian Index of the Environment (PIE) were 1.05 for leisure and 1.04 for shopping purposes [21]. This difference is interpreted based on the indicators included in the walkability index. While the PIE considers the 5Ds of urban form, including density, diversity, design, destination accessibility, and distance to transit [21], the Walkability Score mainly considers destination accessibility [25]. As mentioned earlier, the Walkability Score was assessed based on the accessibility of amenities including grocery stores, restaurants, shopping centers [61]. Therefore, it can be demonstrated that the Walkability Score is an index based on the accessibility of amenities that are more suitable for shopping than for leisure purposes. Second, other neighborhood-level variables had no significant correlation with the odds of nonmotorized trips both in the leisure and shopping models. From Models L–2 and S–2, no variable was significant among land use mix and sidewalk length. This result is different from those of previous studies in which active transport (e.g., walking) and/or physical activity was positively correlated with land use mix [31,64,65]. Meanwhile, some studies showed mixed-use development may not provide a positive effect on walking and cycling in some high-density cities because these cities would reduce the chance for people walking or cycling as they can move easily to another area by public transportation [5,74,75]. This might be applied to Seoul because it is one of the high-density cities [76] and has good public transportation in its neighborhoods. Regarding sidewalk length, this analysis showed a similar result to the previous study that estimated the association between sidewalk length and walking for different trip purposes [66]. It found that neighborhood-based walking for transportation had a positive association with sidewalk length but had no association in recreation walking, which was in line with our research on active transportation for leisure and shopping purposes. Third, the types of individual variables that were significantly associated with the odds of nonmotorized trips differ in the leisure and shopping models. Specifically, older age was positively correlated with the odds of nonmotorized trips for leisure purposes, while female gender had a positive correlation with the odds of nonmotorized trips for shopping purposes. These results can be used to develop various urban policies. For example, older people were more likely to have higher odds of nonmotorized trips for leisure purposes in this study. This finding presents an important issue for further research on age-friendly community design. Environmental factors that promote leisure walking (e.g., greener landscape, well-designed street furniture, wide pedestrian roads, safer pathway) in the development of age-friendly communities may be considered. In addition, a higher Walk Score was correlated to a lower risk of obesity for females in urban areas [43]. As noted from the result, the female gender tends to walk and cycle for shopping purposes; therefore, planning a wider choice of commercial facilities in a walkable and bikeable distance in urban areas or creating a more walkable environment for shopping could induce more physical activity for females. Finally, according to ICC values, the level of walkability of the neighborhood was an important factor in influencing individuals’ odds of nonmotorized trips for shopping purposes. From the results of the multilevel analyses, the proportion accounted for by the Walkability Score in the odds of nonmotorized trips for shopping purposes was about 4.5%. Although the ICC value was high at 9.3% in the leisure model (Model L–1), the Walkability Score was insignificant; therefore, it was not discussed. Additionally, compared to the results of the previous study by Kim et al. [52] (ICC was 2.1% for active commuting model), active trips for shopping had a higher ICC value (4.5%) in this study. This indicates that the Walkability Score is a walkability index that responds more sensitively and effectively to the odds of nonmotorized trips for shopping purposes when compared to commuting purposes.

This study has several limitations; directions for future studies to address them follow. At the outset, even though this study found a significant correlation between the walkability and odds of active transportation for shopping purposes, it showed no correlation between them for leisure models. This may be due to the Walkability Score’s sensitive nature toward walking and cycling for shopping purposes. For further direction of the study, the Walkability Score can be developed and customized based on trip purposes. For example, it will be possible to develop a walkability index, “Walkability Score for Leisure,” where people can search for environmental conditions for leisure purposes in their daily lives by considering the characteristics and contents of each facility that can attract and affect leisure trips. Second, this study examined the correlation of neighborhood walkability with active transportation in Seoul. The city of Seoul is promoting policies regarding walking and cycling, and the neighborhood environment is being developed toward a more walkable environment [51]. Future research will be able to experiment with other cities in Korea, which could help identify practical policies in urban design and transportation planning that can be used across the country. Third, since we focused on active transportation for noncommuting trips, this study combined walking and cycling into nonmotorized transportation. However, walkers and cyclists can have different characteristics of shopping and leisure trips. In a further study, an analysis comparing walking and cycling behavior on each trip’s purposes could be discussed. Fourth, the mean ages of the participants for leisure and shopping purposes were relatively high, corresponding to middle-aged and older adults. This can reflect that retired people may have more time for recreational and shopping activities, and thus they are likely to spend their time on the active mode of the trip. Nevertheless, the older age of respondents remains a limitation. Future studies need to properly extract samples and use them for analysis so that respondents are not biased at a specific age group. Finally, this study is a cross-sectional design that cannot identify causal relationship over time. Cross-sectional analysis cannot identify whether individuals walk frequently because of its walkable environmental conditions or whether the individuals who walk a lot more choose to live in walkable neighborhoods. For further study, a longitudinal analysis can be performed to examine the causality between walkable environment and active transportation.

Despite these limitations, this study is significant in several aspects. First, since the correlation of environmental walkability and active trips was analyzed using a multilevel logistic model, factors from both individual and neighborhood levels were taken into account. Although the ICC values were generally low at 4.5% in Model S–1 and 4.7% in Model S–2, it is suggested that multilevel modeling is required when dealing with nested data [73]. That is, because individual-level variables were nested within the neighborhood, the multilevel analysis allowed an examination of the impact of multilevel factors on the dependent variable (active transportation). Second, there are few studies that comprehensively examined active transportation for both leisure and shopping purposes. In this study, we compared the probability of active transportation (i.e., walking and cycling) for each travel purpose (i.e., leisure and shopping purposes) depending on the Walkability Score. Based on our findings, a tailored policy guideline can be provided based on trip purposes. Finally, we found that the Walkability Score, measured in Seoul, had a significant validity for examining the odds of active transportation for shopping purposes. However, this study demonstrated that more variables should be considered when assessing active transportation, especially for leisure purposes, as they affect not just accessibility to local destinations but also other urban form factors such as density, diversity, and design, and the quality of urban environments. From the findings of this study, policymakers and researchers from the urban and transportation planning and public health fields can obtain a comprehensive understanding of enhancing active travel in leisure and shopping, based on the primary concerns.

## 6. Conclusions

Walking and cycling have attracted the attention of urban planners and policymakers not only as a means of sustainable transport but also to boost individuals’ physical activity levels [5]. The built environment plays a crucial role in promoting active transportation, and various efforts have been made to measure the correlation between neighborhood walkability and active trips. Meanwhile, it is often said that a walkable neighborhood environment can promote the use of active transportation in noncommuting trips [30]. Accordingly, this study identified the correlation between neighborhood walkability and active transportation for leisure and shopping purposes. The results of this study showed that the association between walkability and active transportation varies depending on trip purposes. The results remind urban policymakers of the need to differentiate policy remedies while promoting active transportation. Based on this study, various policy suggestions for promoting active transportation were established, and it is expected that citizens will be encouraged to walk and cycle and increase physical activity to enjoy a better quality of life.

## Figures and Tables

**Table 1:** Measurement, data source, and descriptive statistics of variables.

Variable	Measurement	Data Source
*Dependent Variable*		
Travel mode	Binary: 0 = Motorized mode, 1 = Nonmotorized mode	Household Travel Diary Survey from the Korea Transport Database [54]
*Individual Variables (Level 1)*		
Age	Continuous: Age	Household Travel Diary Survey from the Korea Transport Database [54]
Gender	Binary: 0 = male, 1 = female	
Income	Ordinal: 1 = less than 1 million won, 2 = 1–2 million won, 3 = 2–3 million won, 4 = 3–5 million won, 5 = 5–10 million won, 6 = more than 10 million won	
Car ownership	Binary: 0 = no, 1 = yes	
*Neighborhood Variables (Level 2)*		
Walkability Score	Continuous: Walkability Score	Kim et al. [61]
Land use mix ^1^	Continuous: 0 (single use)–1 (perfect mixing)	National Spatial Data Infrastructure Portal [67]
Sidewalk length	Continuous: Length of sidewalk per square kilometer	

^1^$\mathrm{Land}\;\mathrm{Use}\;\mathrm{Mix}=-1\left(\overset{n}{\underset{i=1}{\sum }}p_{i}\times \ln\left(p_{i}\right)\right)/\ln\left(n\right)$, where *p*_i_ is the proportion of the land use type of i, i = residential, commercial, industrial, and greenspaces, n = total number of land uses in the mix (=4).

**Table 2:** Descriptive statistics of variables.

Variable	Measurement	Leisure Purpose		Shopping Purpose	
		%	Mean (SD)	%	Mean (SD)
*Dependent Variable*					
Travel mode	Binary:				
	0 = Motorized mode	18.2%		23.2%	
	1 = Nonmotorized mode	81.8%		76.8%	
*Individual Variables (Level 1)*					
Age	Continuous: Age		61.1 (16.7)		53.5 (15.1)
Gender	Binary:				
	0 = male	38.7%		9.1%	
	1 = female	61.4%		90.9%	
Income	Ordinal: Household income level		4 ^1^		3 ^2^
Car ownership	Binary:				
	0 = no	45.2%		36.7%	
	1 = yes	54.8%		63.3%	
*Neighborhood Variables (Level 2)*					
Walkability Score	Continuous: Walkability Score		67.55(9.0)		67.71 (9.8)
Land use mix ^3^	Continuous: 0 (single use)–1 (perfect mixing)		0.52(0.3)		0.53 (0.3)
Sidewalk length ^3^	Continuous: Length of sidewalk per square kilometer		1.99(0.6)		1.95 (0.6)

^1^ This is a median value and it corresponds to 3–5 million won, ^2^ this is a median value and it corresponds to 2–3 million won, ^3^ square root-transformed, SD: standard deviation.

**Table 3:** Results of the multilevel logit regression analyses for estimating environmental correlates of active transportation in leisure and shopping purposes.

Variable	Odds of Nonmotorized Trip for Leisure Purpose								Odds of Nonmotorized Trip for Shopping Purpose							
	Model L–1				Model L–2				Model S–1				Model S–2			
	OR	*p-*Value	95% CI		OR	*p-*Value	95% CI		OR	*p-*Value	95% CI		OR	*p-*Value	95% CI	
			Lower	Upper			Lower	Upper			Lower	Upper			Lower	Upper
Intercept.	1.662	0.266	0.766	2.557	1.540	0.375	0.587	2.493	0.879	0.785	−0.051	1.809	0.871	0.789	−0.138	1.881
*Individual Variables (Level 1)*																
Age	1.012 ***	<0.001	1.008	1.017	1.013 ***	0.000	1.008	1.017	1.004	0.227	0.997	1.011	1.003	0.374	0.996	1.010
Gender (reference: male)	1.043	0.600	0.885	1.202	1.050	0.547	0.892	1.208	1.755 ***	<0.001	1.473	2.036	1.735 ***	0.000	1.444	2.026
Income	1.044	0.248	0.971	1.117	1.043	0.263	0.969	1.116	0.996	0.925	0.904	1.087	0.983	0.731	0.886	1.080
Car Ownership (reference: no)	0.519 ***	<0.001	0.301	0.736	0.523 ***	0.000	0.306	0.739	0.646 ***	<0.001	0.407	0.885	0.652 ***	0.001	0.410	0.894
*Neighborhood Variables (Level 2)*																
Walkability Score	1.009	0.145	0.997	1.020	1.009	0.178	0.996	1.022	1.015 *	0.013	1.003	1.026	1.018 **	0.008	1.005	1.031
Land use mix ^1^					1.082	0.699	0.681	1.483					0.944	0.789	0.518	1.369
Sidewalk length ^1^					1.000	1.000	0.797	1.203					0.949	0.646	0.727	1.172
ICC	9.3%				9.2%				4.5%				4.7%			
N	5742								3722							

*** *p* < 0.001, ** *p* < 0.01, * *p* < 0.05; ^1^ square root-transformed; OR = Odds Ratio; CI = Confidence Interval; ICC = Intraclass Correlation Coefficient.
